# Testing the Treatment Integrity of the Managing Cancer and Living Meaningfully Psychotherapeutic Intervention for Patients With Advanced Cancer

**DOI:** 10.3389/fpsyg.2020.561997

**Published:** 2020-12-03

**Authors:** Susan Koranyi, Rebecca Philipp, Leonhard Quintero Garzón, Katharina Scheffold, Frank Schulz-Kindermann, Martin Härter, Gary Rodin, Anja Mehnert-Theuerkauf

**Affiliations:** ^1^Department of Medical Psychology and Medical Sociology, Section of Psychosocial Oncology, University Medical Center Leipzig, Leipzig, Germany; ^2^Department of Medical Psychology, University Medical Center Hamburg-Eppendorf, Hamburg, Germany; ^3^Department of Supportive Care, Princess Margaret Cancer Centre, University Health Network, Toronto, ON, Canada

**Keywords:** treatment integrity, therapists’ adherence, treatment differentiation, managing cancer and living meaningfully, psycho-oncological intervention, advanced cancer

## Abstract

**Introduction:**

The Managing Cancer and Living Meaningfully (CALM) therapy for patients with advanced cancer was tested against a supportive psycho-oncological counseling intervention (SPI) in a randomized controlled trial (RCT). We investigated whether CALM was delivered as intended (therapists’ adherence); whether CALM therapists with less experience in psycho-oncological care show higher adherence scores; and whether potential overlapping treatment elements between CALM and SPI can be identified (treatment differentiation).

**Methods:**

Two trained and blinded raters assessed on 19 items four subscales of the Treatment Integrity Scale covering treatment domains of CALM (SC: Symptom Management and Communication with Health Care Providers; CSR: Changes in Self and Relationship with Others; SMP: Spiritual Well-being and Sense of Meaning and Purpose; FHM: Preparing for the Future, Sustaining Hope and Facing Mortality). A random sample of 150 audio recordings (75 CALM, 75 SPI) were rated on a three-point Likert scale with 1 = “adherent to some extent,” 2 = “adherent to a sufficient extent,” 3 = “very adherent.”

**Results:**

All 19 treatment elements were applied, but in various frequencies. CALM therapists most frequently explored symptoms and/or relationship to health care providers (SC_1: n__applied_ = 62; 83%) and allowed expression of sadness and anxiety about the progression of disease (FHM_2: n__applied_ = 62; 83%). The exploration of CALM treatment element SC_1 was most frequently implemented in a satisfactory or excellent manner (n__sufficient or very adherent_ = 34; 45%), whereas the treatment element SMP_4: Therapist promotes acknowledgment that some life goals may no longer be achievable (n__sufficient or very adherent_ = 0; 0%) was not implemented in a satisfactory manner. In terms of treatment differentiation, no treatment elements could be identified which were applied significantly more often by CALM therapists than by SPI therapists.

**Conclusion:**

Results verify the application of CALM treatment domains. However, CALM therapists’ adherence scores indicated manual deviations. Furthermore, raters were not able to significantly distinguish CALM from SPI, implying that overlapping treatment elements were delivered to patients.

## Introduction

In order to draw internally valid conclusions about treatment effects in clinical trials, it is important to monitor treatment fidelity (Weck et al., 2011). The Treatment Fidelity Workgroup of the NIH Behavior Change Consortium proposes five areas of treatment fidelity: study design, provider training, treatment receipt, enactment of treatment skills, and treatment delivery (Bellg et al., 2004). Whether a treatment is delivered as intended (treatment integrity; Yeaton and Sechrest, 1981; Moncher and Prinz, 1991; Perepletchikova, 2011), can be differentiated in three aspects: therapists’ adherence – the extent to which therapists deliver a treatment according to the treatment manual (Weck et al., 2011); therapists’ competence – the amount of therapeutic skill and quality in the implementation of therapeutic techniques (Sharpless and Barber, 2009); and treatment differentiation – how the treatment of interest can be distinguished from other forms of treatment in case multiple treatments conditions are tested (Waltz et al., 1993; Barber et al., 2007; Weck et al., 2011). Thus, treatment integrity is an essential precondition for the evaluation of interventions in psycho-oncological research. Assuring treatment integrity is also important for dissemination of evidence-based practices and quality improvement of services (Perepletchikova, 2011).

A current systematic review (Teo et al., 2019) examined 68 randomized controlled trials (RCTs) of psycho-oncological interventions for advanced cancer patients. Most studies (87%) implemented one or more measures to ensure consistency in treatment delivery through treatment manuals, supervision, and/or review of audio recorded therapy sessions. However, quantitative data on treatment integrity were reported in only one of those RCTs, more precisely, in the context of an RCT evaluating the Managing Cancer and Living Meaningfully (CALM) therapy (Rodin et al., 2018). CALM was developed by Gary Rodin and colleagues and has been implemented in the Canadian health care system over a period of 10 years (Nissim et al., 2012; Lo et al., 2014, 2015). Since then CALM has been successfully evaluated, showing greater reduction of depressive symptoms, stronger abilities to communicate with health care providers, and a greater sense of hope and meaning in life when compared to usual care (Rodin et al., 2018). Recently, the efficacy of CALM therapy was tested against a non-manualized supportive psycho-oncological counseling intervention (SPI) within the German health care system (Mehnert et al., 2020). Within this RCT, CALM therapy was associated with reduction in depressive symptoms over time but this improvement was not statistically significant different than that obtained within SPI group (Mehnert et al., 2020). In the German CALM RCT, we monitored and measured treatment integrity according to the suggestions in the literature (Bellg et al., 2004; Perepletchikova et al., 2009; Weck et al., 2011): The CALM therapy manual (Hales et al., 2010) contains semi-structured descriptions of the therapeutic content to be addressed, along with the techniques to be used. According to the stage model for the development of psychotherapy manuals (Carroll and Nuro, 2002), the CALM manual can be classified as a stage II manual, which specifies training and supervision, treatment elements, and evaluation of the treatment process. It includes external aspects, such as the number of therapy sessions, length of a session, and the qualification of therapists, as well as internal aspects, such as general therapeutic skills important to deliver CALM therapy (e.g., empathic understanding of patient’s experience; ability to appropriately modulate the emotional state of the patient; ability to shift between supportive, exploratory, and problem-solving therapeutic frames when necessary). Most importantly, the manual offers a detailed description of the contents of CALM therapy: the rationale that patients with advanced cancer benefit from psycho-oncological treatment when the four dimensions (1) *Symptom Management and Communication with Health Care Providers*, (2) *Changes in Self and Relationship with Others*, (3) *Spiritual Well-being and the Sense of Meaning and Purpose*, and (4) *Preparing for the Future, Sustaining Hope and Facing Mortality* (Hales et al., 2010).

In the present study, we aim to evaluate treatment integrity within the German CALM RCT by examining therapists’ adherence and the impact of CALM therapists’ experience on their adherence to CALM manual. Based on clinical observations we hypothesize that CALM therapists with less experience in psycho-oncological care have a higher motivation to follow manual guidelines, which in turn is associated with higher adherence scores. We further aim to test whether both treatment conditions (CALM vs. SPI) were different from one another regarding the application of treatment elements (treatment differentiation).

The objectives of our study are (1) to verify the application of CALM manual interventions within CALM therapy sessions, (2) to quantify the extent to which CALM interventions were delivered as intended by CALM therapists, (3) to test whether CALM therapists’ experience is associated with the frequency of applied treatment elements, and (4) to check whether blinded raters can distinguish the two treatment conditions (CALM vs. SPI).

## Materials and Methods

### Study Design, Participants, and Procedures

We assessed treatment integrity using the audio recordings of therapy sessions throughout the CALM RCT (clinicaltrials.gov NCT02051660), which was conducted between August 2013 and January 2017 (Mehnert et al., 2020). A total of 206 patients were recruited in two German University Medical Centers (Hamburg and Leipzig). The study protocol (Mehnert et al., 2020) was approved by local ethics committees in both study centers (Hamburg reference number: PV4435; Leipzig reference number: 143–14–14042014). Data for the present study were obtained from August 2013 until January 2016.

We included patients who were diagnosed with a malignant solid tumor [Union internationale contre le cancer (UICC) stage III or IV], ≥18 years, fluent in German, and who reported ≥ 9 on the depression module of the Patient Health Questionnaire (PHQ-9, Löwe et al., 2004) and/or ≥ 5 on the Distress Thermometer (DT, Mehnert et al., 2006). We excluded patients who showed deficits in communication, a score < 20 on the Short Orientation-Memory-Concentration test (Katzman et al., 1983; Wade and Vergis, 1999), a level < 70 on the Karnofsky index (Evans and Mccarthy, 1985; Kaasa and Loge, 2003), or suicidal ideation. We further excluded patients in case they received parallel psychotherapy. All patients provided written informed consent prior to participation and could withdraw their consent without having any disadvantage in their medical or psycho-oncological treatment. After randomization, patients were not informed about their allocated treatment condition (single-blinded trial).

### CALM Therapy

Cancer and Living Meaningfully therapy consists of four therapeutic key domains which are supposed to be addressed during treatment (Hales et al., 2010):

(1) *SC: Symptom Management and Communication with Health Care Providers*: The first domain focuses on the cooperation and improvement of communication with health care providers and offers help regarding medical decision-making to ensure best care and control of symptoms.
(2) *CSR: Changes in Self and Relations with Close Others:* The second domain focuses on patients’ adjustment of self-esteem and identity due to cancer-related changes and takes into account relationships with close others, especially sustaining and providing required care and support.
(3) *SMP: Spirituality, Sense of Meaning, and Purpose:* The third domain focuses on understanding the individual meaning of suffering and dying, and includes an evaluation of priorities and goals facing an advanced disease.
(4) *FHM: Preparing for the Future, Sustaining Hope and Facing Mortality:* The last domain deals with the acknowledgment of anticipatory fears, balancing life and death, planning advanced treatment, and preparing for the process of dying.

The CALM therapy manual (Hales et al., 2010) was translated into German by the German study group following state-of-the art procedures resulting in a German CALM therapy manual – research version.

### Non-manualized Supportive Psycho-Oncological Counseling Intervention (SPI)

Supportive Psycho-Oncological Counseling Intervention patients received a non-manualized SPI, which is the usual care for patients reporting heightened mental distress in both study centers. Like CALM therapy, patients in the SPI group received three to eight individual therapy sessions lasting 50 min each over a period of 6 months. The SPI integrative approach helps patients to deal with distressing emotions, to reinforce pre-existing resources, and to promote adaptive coping with cancer (Lederberg and Holland, 2011). It contains components of psychoeducation, psycho-oncological counseling (e.g., strategies to manage overwhelming emotions), as well as crisis intervention. Moreover, it promotes learning and problem-solving using cognitive-behavioral and psychodynamic techniques.

### Therapists

Cancer and Living Meaningfully therapists received the CALM manual and were trained in a 2-day CALM workshop held by CALM-experienced psycho-oncologists who were previously trained by Gary Rodin and colleagues (AM-T and FS-K). Therapists received supervision with a CALM-experienced supervisor every 4 weeks. Therapists did not receive feedback about their individual scoring on the Treatment Integrity Scale during supervision. However, every case was discussed with focus on high treatment integrity. SPI therapists received the same amount of supervision as CALM therapists with non-CALM-trained supervisors and were blinded to the content of the CALM manual.

Therapists were licensed as clinical psychotherapists. For the RCT, we aimed to balance sociodemographic characteristics (age, sex) and therapists’ experience in psycho-oncological care between groups by building pairs of comparable therapists and allocating one in each pair randomly to CALM and to SPI, respectively. For the present study, we randomly selected therapy sessions of 10 CALM therapists and eight SPI therapists in order to rate therapists’ adherence and treatment differentiation.

### Measures

Rodin and a group of CALM experts developed a Treatment Integrity Scale adapted from Spiegel and Spira (1991). The Treatment Integrity Scale consists of 32 items on eight subscales (Rodin et al., 2018): *Therapeutic process subscales*: (1) Therapeutic Relationship; (2) Modulating Affect; (3) Shifting Frame; (4) Interpretations; *Therapeutic content subscales*: (5) Symptom Management and Communication with Health Care Providers; (6) Changes in Self and Relations with Close Others; (7) Spirituality, Sense of Meaning, and Purpose; (8) Preparing for the Future, Sustaining Hope and Facing Mortality. For the present study, we rated the four *Therapeutic content subscales* with the following treatment elements on 19 items:

(1) SC_1: Therapist explores symptoms and/or relationship to health care providers.
(2) SC_2: Therapist encourages better understanding of disease symptoms.
(3) SC_3: Therapist promotes patient’s consideration of treatment options.
(4) SC_4: Therapist encourages patient’s active involvement in medical care and supports communication with health care providers for better symptom control and medical decision-making.
(5) CSR_1: Therapist explores patient’s feelings about his or her life story.
(6) CSR_2: Therapist validates patient’s sense of worth in light of his or her accomplishments in the domains of family, work, and community.
(7) CSR_3: Therapist acknowledges disappointments or regrets that the patient has experienced in those domains.
(8) CSR_4: Therapist explores the relational changes imposed by disease.
(9) CSR_5: Therapist explores fears and anxieties about dependency and loss of autonomy.
(10) CSR_6: Therapist encourages appropriate communication (i.e., support-giving/taking from close others).
(11) SMP_1: Therapist explores the patient’s spiritual beliefs and/or sense of meaning and purpose in life.
(12) SMP_2: Therapist explores personal meaning of suffering and dying.
(13) SMP_3: Therapist evaluates priorities and goals in the face of advanced disease.
(14) SMP_4: Therapist promotes acknowledgment that some life goals may no longer be achievable.
(15) FHM_1: Therapist explores patient’s attitudes toward the future.
(16) FHM_2: Therapist allows expression of sadness and anxiety about the progression of disease.
(17) FHM_3: Therapist explores feelings about death and dying.
(18) FHM_4: Therapist promotes discussion of advanced care planning.
(19) FHM_5: Therapist helps to sustain realistic hope and engagement in life while acknowledging mortality.

Depending on the patient’s individual needs, CALM therapists were free to choose treatment elements. Accordingly, not all 19 items were applied in every therapy session. Thus, raters first had to indicate whether treatment elements were “applied” or “not applied.” After an item was coded as “applied,” the extent of therapists’ adherence had to be estimated on a three-point Likert scale with “1 = adherent to some extent” for an acceptable, but not satisfactory implementation, “2 = adherent to a sufficient extent” for a satisfactory implementation, “3 = very adherent” for an excellent implementation of a CALM therapy technique as described in the CALM manual.

### Audio Recordings, Raters, and Rating Procedure

All therapy sessions throughout the CALM RCT were recorded. We randomly selected therapy sessions from 50 patients (CALM: n = 25, SPI: n = 25), in order to measure treatment integrity in about 25% of the total CALM RCT patient sample which is slightly above the average amount of adherence ratings in previous psycho-oncological intervention studies (Ando et al., 2010; Henry et al., 2010). For each of the 50 patients, we chose the audio recording of the first and the last, as well as of an intermediate therapy session.

All audio recordings were independently rated by two psychologists who both had a master’s degree in clinical psychology. They were not involved in the CALM RCT and were not familiar with any of the CALM or SPI therapists. Both raters were trained in a 2-day CALM workshop (including the training slides and CALM manual).

Both raters were blinded to the objective 4 (treatment differentiation) of the present study: They were instructed to rate the audio recordings “derived from CALM treatment group”; we did not inform raters that half of the audio recordings contained therapy sessions from SPI condition.

### Statistical Analysis

#### Interrater Reliability

During the pilot phase of the present study, we checked interrater reliability for the discrimination between “applied” and “not applied” treatment elements by calculating Cohen’s κ for each item, considering κ ≥ 0.75 as excellent, 0.40–0.75 as moderate, and <0.40 as poor (Fleiss and Cohen, 1973). Second, following the recommendations by Shrout and Fleiss (1979), we calculated ICCs using Model 2 [ICC _(2, 2)_]. If an item had Cohen’s κ ≥ 0.40, ICCs ≥ 0.75 represented good, ICCs between 0.50 and 0.75 moderate, and ICC < 0.50 poor interrater reliability (Koo and Li, 2016).

#### CALM Therapists’ Adherence

In order to verify the application of the CALM manual interventions within CALM therapy sessions (objective 1), we counted the frequencies of “applied” (n__applied_) for each item. To quantify the extent to which CALM interventions were delivered as intended by CALM therapists (objective 2), we summed up frequencies of score 2 “adherent to a sufficient extent” and score 3 “very adherent” ratings (n__sufficiently or very adherent_).

#### CALM Therapists’ Experience in Psycho-Oncological Care

We examined whether frequencies of applied CALM techniques differed with regard to the level of their experience using χ^2^ tests with a significance level of 0.003 corrected for multiple testing according to Bonferroni (objective 3).

#### Treatment Differentiation

To check if the two treatment conditions (CALM vs. SPI) can be significantly distinguished by blinded raters, treatment differentiation was tested using χ^2^ tests with a significance level of *p* = 0.003 corrected for multiple testing according to Bonferroni (objective 4).

Data analyses were conducted using IBM SPSS^®^, Version 26.

## Results

### Patients

Patients of the present study (n = 50) were 70% female with a mean age of 57.4 years (SD = 11.7). UICC III tumor stage was diagnosed in 14%, UICC IV in 86% of the patients with various tumor entities: gastrointestinal (37%), gynecological (20%), breast (12%), lung (10%), urogenital (8%), head or neck (4%), and other (8%). The present random patient sample did not differ statistically significant from the total CALM RCT patient sample with regard to sex (*p* = 0.18), age (*p* = 0.72), UICC tumor stage (*p* = 0.68), or tumor localization (*p* = 0.21).

### Therapists

Therapists were mostly female, with 70% in the CALM group and 75% in the SPI group. CALM and SPI therapists did not differ statistically significant in their mean age (CALM: M = 42.2; SD = 10.9; range = 32–60; SPI: M = 50.8; SD = 10.7; range = 31–64, *p* = 0.11). The range of CALM therapists’ experience in psycho-oncological care was 8–30 years (M = 15.05; SD = 8.3) within the group of CALM therapists and 5–30 years (M = 15.8; SD = 8.7) within the group of SPI therapists.

### Interrater Reliability

During the pilot phase, interrater reliability was calculated for 26 audio recordings. For items with Cohen’s κ ≥ 0.40, ICC was considered as good for only one item (CSR_1: Therapist explores patient’s feelings about his or her life story, ICC = 0.771), moderate for 10 items (lowest ICC = 0.504 for SMP_2: Therapist explores personal meaning of suffering and dying; highest ICC = 0.744 for FHM_4: Therapist promotes discussion of advanced care planning), and poor for four items (lowest ICC = 0.368 for SMP_3: Therapist evaluates priorities and goals in the face of advanced disease; highest ICC = 0.476 for SC_3: Therapist promotes patient’s consideration of treatment options; see Table 1). Thus, after the pilot phase, we decided that raters code audio recordings independently first and subsequently compare their assessments item by item for each therapy session. In case of deviations, raters reviewed the audio material and a consensus discussion was carried out. Thus, all statistical analyses were carried out with the consensus score of both raters.

**TABLE 1:** Interrater reliability.

	*Interrater reliability*	
	Two raters (26 pilot-ratings)	
	κ	ICC
SC_1: Therapist explores symptoms and/or relationship to health care providers.	0.506	0.682
SC_2: Therapist encourages better understanding of disease symptoms.	0.510	0.585
SC_3: Therapist promotes patient’s consideration of treatment options.	0.528	0.476
SC_4: Therapist encourages patient’s active involvement in medical care and supports communication with health care providers for better symptom control and medical decision-making.	0.709	0.658
CSR_1: Therapist explores patient’s feelings about their life story.	0.574	0.771
CSR_2: Therapist validates patient’s sense of worth in light of their accomplishments in the domains of family, work, and community.	0.503	0.384
CSR_3: Therapist acknowledges disappointments or regrets that the patient has experienced in those domains.	0.466	0.481
CSR_4: Therapist explores the relational changes imposed by disease.	0.646	0.553
CSR_5: Therapist explores fears and anxieties about dependency and loss of autonomy.	0.342	–
CSR_6: Therapist encourages appropriate communication (i.e., support-giving/taking from close others).	0.421	0.628
SMP_1: Therapist explores the patient’s spiritual beliefs and/or sense of meaning and purpose in life.	0.633	0.695
SMP_2: Therapist explores personal meaning of suffering and dying.	0.549	0.504
SMP_3: Therapist evaluates priorities and goals in the face of advanced disease.	0.445	0.368
SMP_4: Therapist promotes acknowledgment that some life goals may no longer be achievable.	−0.019	–
FHM_1: Therapist explores patient’s attitudes toward the future.	0.199	–
FHM_2: Therapist allows expression of sadness and anxiety about the progression of disease.	0.326	–
FHM_3: Therapist explores feelings about death and dying.	0.408	0.514
FHM_4: Therapist promotes discussion of advanced care planning.	0.604	0.744
FHM_5: Therapist helps to sustain realistic hope and engagement in life while acknowledging mortality.	0.726	0.695

*κ, Cohen’s kappa; ICC, intraclass correlations.*

### CALM Therapists’ Adherence

All 19 treatment elements were applied, but in various frequencies (Table 2). Three items (SC_1: Therapist explores symptoms and/or relationship to health care providers, CSR_6: Therapist encourages appropriate communication, FHM_2: Therapist allows expression of sadness and anxiety about the progression of disease) were applied in > 75% of CALM sessions. The most frequently applied treatment elements within CALM sessions were SC_1: Therapist explores symptoms and/or relationship to health care providers (n__applied_ = 62; 83%) and FHM_2: Therapist allows expression of sadness and anxiety about the progression of disease (n__applied_ = 62; 83%).

**TABLE 2:** Frequencies of CALM therapists’ adherence; tests of differences in CALM therapists’ adherence depending on CALM therapists’ experience; treatment differentiation between CALM and SPI.

	*CALM therapists’ adherence*			*CALM therapists’ experience*	*Treatment differentiation*	
	CALM sessions				SPI session	
	Range (min/max)	n__sufficient or very adherent (%)_	n___applied_ (%)	χ^2^ test _(first levelvs. second level of *experience*)_	n__applied_ (%)	χ^2^ Test _(*CALM vs. SPI*)_
SC_1: Therapist explores symptoms and/or relationship to health care providers.	1/3	34 (45)	62 (83)	χ^2^ (1, N = 75) = 10.230; *p = 0.001	53 (71)	χ^2^ (1, N = 150) = 5.430; *p* = 0.020
SC_2: Therapist encourages better understanding of disease symptoms.	1/2	2 (3)	22 (29)	χ^2^ (1, N = 75) = 1.320; *p* = 0.251	10 (13)	χ^2^ (1, N = 150) = 6.567; *p* = 0.010
SC_3: Therapist promotes patient’s consideration of treatment options.	1/3	2 (3)	12 (16)	χ^2^ (1, N = 75) = 2.687; *p* = 0.101	11 (15)	χ2 (1, N = 150) = 0.134; *p* = .715
SC_4: Therapist encourages patient’s active involvement in medical care and supports communication with health care providers for better symptom control and medical decision-making.	1/3	13 (17)	32 (43)	χ^2^ (1, N = 75) = 1.886; *p* = 0.170	30 (40)	χ^2^ (1, N = 150) = 0.367; *p* = 0.545
CSR_1: Therapist explores patient’s feelings about their life story.	1/3	23 (31)	44 (59)	χ^2^ (1, N = 75) = 1.429; *p* = 0.232	52 (69)	χ^2^ (1, N = 150) = 0.857; *p* = 0.355
CSR_2: Therapist validates patient’s sense of worth in light of their accomplishments in the domains of family, work, and community.	1/2	5 (7)	20 (27)	χ^2^ (1, N = 75) = 0.479; *p* = 0.489	36 (48)	χ^2^ (1, N = 150) = 6.001; *p* = 0.014
CSR_3: Therapist acknowledges disappointments or regrets that the patient has experienced in those domains.	1/3	16 (21)	38 (51)	χ^2^ (1, N = 75) = 1.168; *p* = 0.280	42 (56)	χ^2^ (1, N = 150) = 0.093; *p* = 0.760
CSR_4: Therapist explores the relational changes imposed by disease.	1/3	8 (11)	35 (47)	χ^2^ (1, N = 75) = 1.606; *p* = 0.205	30 (40)	χ^2^ (1, N = 150) = 1.232; *p* = 0.267
CSR_5: Therapist explores fears and anxieties about dependency and loss of autonomy.	1/2	1 (1)	9 (12)	χ^2^ (1, N = 75) = 0.019; *p* = 0.891	9 (12)	χ^2^ (1, N = 150) = 0.015; *p* = 0.904
CSR_6: Therapist encourages appropriate communication (i.e., support-giving/taking from close others).	1/3	26 (35)	55 (73)	χ^2^ (1, N = 75) = 0.567; *p* = 0.451	50 (67)	χ^2^ (1, N = 150) = 1.933; *p* = 0.164
SMP_1: Therapist explores the patient’s spiritual beliefs and/or sense of meaning and purpose in life.	1/2	2 (3)	13 (17)	χ^2^ (1, N = 75) = 3.263; *p* = 0.071	7 (9)	χ^2^ (1, N = 150) = 2.464; *p* = 0.116
SMP_2: Therapist explores personal meaning of suffering and dying.	1/2	1 (1)	14 (19)	χ^2^ (1, N = 75) = 0.082; *p* = 0.775	9 (12)	χ^2^ (1, N = 150) = 1.619; *p* = 0.203
SMP_3: Therapist evaluates priorities and goals in the face of advanced disease.	1/2	11 (15)	29 (39)	χ^2^ (1, N = 75) = 1.445; *p* = 0.229	29 (39)	χ^2^ (1, N = 150) = 0.067; *p* = 0.795
SMP_4: Therapist promotes acknowledgment that some life goals may no longer be achievable.	1/1	0 (0)	3 (4)	χ^2^ (1, N = 75) = 3.589; *p* = 0.058	2 (3)	χ^2^ (1, N = 150) = 0.266; *p* = 0.606
FHM_1: Therapist explores patient’s attitudes toward the future.	1/2	3 (4)	24 (32)	χ^2^ (1, N = 75) = 3.644; *p* = 0.056	13 (17)	χ^2^ (1, N = 150) = 5.158; *p* = 0.023
FHM_2: Therapist allows expression of sadness and anxiety about the progression of disease.	1/3	28 (37)	62 (83)	χ^2^ (1, N = 75) = 0.543; *p* = 0.461	60 (80)	χ^2^ (1, N = 150) = 1.213; *p* = 0.271
FHM_3: Therapist explores feelings about death and dying.	1/3	6 (8)	26 (35)	χ^2^ (1, N = 75) = 1.068; *p* = 0.301	15 (20)	χ^2^ (1, N = 150) = 4.912; *p* = 0.027
FHM_4: Therapist promotes discussion of advanced care planning.	1/3	2 (3)	10 (13)	χ^2^ (1, N = 75) = 0.055; *p* = 0.815	4 (5)	χ^2^ (1, N = 150) = 3.202; *p* = 0.074
FHM_5: Therapist helps to sustain realistic hope and engagement in life while acknowledging mortality.	1/3	5 (7)	13 (17)	χ^2^ (1, N = 75) = 5.854; *p* = 0.0155	3 (4)	χ^2^ (1, N = 150) = 7.611; *p* = 0.006

CALM therapists’ adherence: n__sufficiently or very adherent_ = frequency of CALM therapists’ adherence assessments with score 2 = “adherent to a sufficient extent” or score 3 = “very adherent”; n_applied = frequencies of applied assessments; CALM therapists’ experience: χ^2^ test of differences in frequencies of applied treatment elements between CALM therapists with first level and second level of experience; Treatment differentiation: χ^2^ test of differences in frequencies of applied treatment elements between CALM and SPI therapists. *p < 0.003.

Four out of 19 CALM treatment elements (SC_1: Therapist explores symptoms and/or relationship to health care providers, CSR_1: Therapist explores patient’s feelings about his or her life story, CSR_6: Therapist encourages appropriate communication, FHM_2: Therapist allows expression of sadness and anxiety about the progression of disease) were administered to a sufficient or very adherent extent in at least 30% of CALM therapy sessions. The highest percentage of “adherent to a sufficient extent” or “very adherent” ratings was obtained for item SC_1: Therapist explores symptoms and/or relationship to health care providers (n__sufficient or very adherent_ = 34; 45%), lowest for SMP_4: Therapist promotes acknowledgment that some life goals may no longer be achievable (n__sufficient or very adherent_ = 0).

### CALM Therapists’ Experience in Psycho-Oncological Care

We classified CALM therapists according to their experience in psycho-oncological care by calculating 50% percentiles (first level: 8–11 years; second level: 12–30 years; Median = 11 years; see Table 3 for therapists’ allocation to levels of experience). Table 2 contains the results of χ^2^ test of differences in frequencies of applied treatment elements between CALM therapists with first level and second level of experience. CALM therapists with second level experience did statistically significant apply more frequent SC_1: Therapist explores symptoms and/or relationship to health care providers (*p* = 0.001).

**TABLE 3:** Individual CALM therapist’s experience level.

Therapist	n__patients_ per therapist (%)	Years of therapist’s experience	Individual therapist’s experience level*
1	4 (16.6)	9	First
2	2 (8.3)	8,5	First
3	4 (16.6)	19	Second
4	2 (8.3)	30	Second
5	1 (4.2)	15	Second
6	3 (12.5)	29	Second
7	1 (4.2)	10	First
8	3 (12.5)	8	First
9	3 (12.5)	12	Second
10	1 (4.2)	10	First

*50% percentiles: First: therapists’ experience in psycho-oncological care from 8 to 11 years; Second: therapists’ experience in psycho-oncological care from 12 to 30 years.

### Treatment Differentiation

There were no statistically significant differences between the two treatment conditions on a single item basis regarding the proportion of applied treatment elements in CALM compared to SPI therapy sessions (Table 2).

## Discussion

In the present research, we aimed to evaluate treatment integrity within the German CALM RCT. We measured CALM therapists’ adherence and tested whether CALM therapists’ experience was associated with their adherence to the manual. In addition, we tested whether both treatment conditions (CALM vs. SPI) contained overlapping treatment elements.

Results verified the application of CALM manual interventions. All 19 CALM treatment elements were addressed within CALM therapy sessions, yet to different extents. Furthermore, none of the CALM treatment elements seemed inapplicable. We also found that not all of the 19 CALM treatment elements were administered in every CALM therapy session. This selection of treatment elements is prescribed in the manual; or more specifically, CALM therapists are instructed to choose in each therapy session the suitable treatment element depending on the patient’s needs.

Overall, results of CALM therapists’ adherence indicate deviations from the CALM manual. In contrast, previous CALM trials obtained satisfactory to excellent treatment integrity ratings referring to CALM therapy (Rodin et al., 2018), indicating only marginal deviations from CALM manual. Therapists delivering CALM treatment within the RCT of Rodin et al. (2018) consistently received feedback on their scoring on the Treatment Integrity Scale during weekly supervision based on case discussion and review of session audio recordings, whereas we did not provide feedback according to individual scores, which might be associated to therapists’ low to moderate levels of adherence. The efficacy of feedback regarding the improvement of therapeutic competencies in cognitive behavioral therapy has recently been shown in a randomized controlled pilot study comparing a competence feedback group against a control group of therapists receiving no additional qualitative and quantitative feedback beyond a regular supervision (Weck et al., 2017). Further research based on a randomized controlled design is required to test whether concomitant feedback improves therapists’ adherence likewise.

The frequencies of applied CALM treatment elements were moderate as well. One reason might be that raters judged only three therapy sessions instead of the total number of therapy sessions per patient. Audio recordings included the first and the last therapy session which represent both a special stage in the course of therapy. The first session is mainly characterized by attempts to build a therapeutic relationship, whereas the last CALM therapy session addresses the termination of treatment while providing still an open-door policy to terminally ill patients and expressing that the therapist has not “given up” the patient. Thus, it is unlikely that CALM therapists applied in the first and last session as much content-related CALM treatment elements as in the middle sessions which in turn might have led to an underestimation of frequency scores. It has been shown in psychotherapy research that 5–10 therapy sessions per patient are needed to achieve sufficient generalizability for ratings of therapists’ adherence and competence (Dennhag et al., 2012). However, due to various reasons of attrition (i.e., patients deceased or had poor physical condition), the average number of CALM sessions was 5, and 54% of CALM patients received at least five sessions of treatment at all (Mehnert et al., 2020). Nevertheless, recommendations for future studies imply the consideration and selection of all treatment sessions in order to cover the entire course of therapy, and to examine the suitability of rating session segments instead of the whole therapy sessions for the evaluation of treatment integrity in psycho-oncological intervention studies. Previous research showed that adherence ratings from segments of the middle third of therapy have a comparable reliability to entire session-based ratings (Weck et al., 2014).

Most of the delivered CALM treatment elements addressed the topic of disease related changes in self and in relationships with others (*Changes in Self and Relations with Close Others* domain), whereas treatment elements dealing with patients’ spiritual well-being and the sense of meaning and purpose (*Spirituality, Sense of Meaning, and Purpose* domain) were less commonly applied in our study. The therapeutic goals of the *Spirituality, Sense of Meaning, and Purpose* domain are to aid in evaluation of priorities and goals in the face of advanced disease and to facilitate a rather active approach to the dying experience (Hales et al., 2010). However, it might have been unfamiliar for therapists to specifically address spirituality due to the fact that dealing with spiritual contents is not integrated in common psycho-oncological or psychotherapeutic education and training in Germany.

Results also revealed that three of the CALM treatment elements were administered in a considerable number of therapy sessions, i.e., the exploration of disease related symptoms and the relationship to health care providers, the encouragement of an appropriate communication with relevant others, and allowing expression of sadness and anxiety about the progression of disease. These treatment elements were applied in at least 75% of therapy sessions and were satisfactorily delivered as it was intended in the manual. In contrast, some CALM elements were only marginally administered, i.e., therapist very rarely promoted acknowledgment that some life goals may no longer be achievable. One possible explanation might be that this specific end-of-life issue and especially the acknowledgment of impending mortality is the most difficult one to implement in CALM therapy, possibly accompanied by denial and defense mechanisms of patients and CALM therapists, respectively.

To our knowledge, this is the first study examining the relationship between therapists’ experience in psycho-oncological care and therapists’ adherence. We obtained differences in frequencies of applied treatment elements depending on therapists’ level of experience. Therapists with more than 11 years of therapeutic experience explored more often symptoms and/or relationship to health care providers than therapists with less experience.

Properly one of the most interesting results is that the treatment conditions (CALM vs. SPI) of the CALM RCT did not differ significantly from each other with regard to the frequency of the applied treatment elements. This low level of treatment differentiation implies that on average raters could not distinguish CALM from SPI therapy and that SPI and CALM therapists delivered similar treatment elements. CALM therapy draws upon several manualized effective psychotherapeutic interventions that have been developed to address distress in patients with advanced cancer. It is based on empirical research results, clinical observations, and theoretical concepts of supportive-expressive, existential, and psychodynamic therapy as well as stress- and problem-solving trainings. Therefore, it is reasonable that SPI therapists who worked in psycho-oncological care centers for several years deliver overlapping treatment elements to CALM therapy.

### Limitations

A shortcoming of this study is that calculations of internal consistency were not possible, since most items were not applied in each session. In addition, another limitation of the present study includes the insufficient interrater reliability. Therefore, the interpretation of the data should be done under consideration of the restriction that raters had difficulties using the rating instrument in a concordant manner. We attenuated this problem by using a consensus version of data after discussing deviations with both raters, which was a time-consuming process and related to higher costs. The treatment integrity rating instrument should only be used in future CALM trials, if an evaluation of psychometric properties was carried out and a subsequent adaption of items followed.

Furthermore, we cannot exclude possible selection and confounding biases due to our approach and the relatively small sample size. We also cannot answer the question of whether patient characteristics (such as sex, age, or level of symptoms) might have an influence on therapists’ adherence and thereby on the implementation of the CALM intervention since we did not follow a stratified approach.

Finally, our study may contribute to a more transparent evaluation of treatment integrity in future psycho-oncological intervention research. Okuyama et al. (2017) reported in their meta-analysis of psychotherapeutic interventions for depression among patients with incurable cancer that only two out of 13 RCTs used direct assessment methods, such as information from audio tapes or video tapes evaluated by independent judges which is preferable to indirect methods (i.e., supervision, therapist’s self-report) in order to monitor treatment integrity appropriately.

In future research, the correlation between therapists’ adherence and treatment outcome could be addressed using CALM RCT audio recordings. The question arises whether adherent and competently conducted CALM therapies also lead to improved treatment outcome.

## Data Availability Statement

The raw data supporting the conclusions of this article will be made available by the authors, without undue reservation.

## Ethics Statement

The studies involving human participants were reviewed and approved by Ethik-Kommission an der Medizinischen Fakultät der Universität Leipzig; Ethik-Kommission der Ärztekammer Hamburg. The patients/participants provided their written informed consent to participate in this study.

## Author Contributions

SK, LQ, and AM-T contributed to conception and design. AM-T and MH provided administrative support. AM-T, FS-K, and GR contributed to provision of study materials and patients. SK, LQ, KS, and RP contributed to collection and assembly of data. SK and RP contributed to data analysis and interpretation. All authors wrote the manuscript and gave final approval of the manuscript.

## Conflict of Interest

The authors declare that the research was conducted in the absence of any commercial or financial relationships that could be construed as a potential conflict of interest.
